# A phase I/IIa study of the mRNA based cancer vaccine CV9103 prepared with the RNActive technology results in distinctly longer survival than predicted by the Halabi Nomogram which correlates with the induction of antigen-specific immune responses

**DOI:** 10.1186/2051-1426-1-S1-P219

**Published:** 2013-11-07

**Authors:** Karl-Josef Kallen, Ulrike Gnad-Vogt, Birgit Scheel, Gerd Rippin, Arnulf Stenzl

**Affiliations:** 1 CureVac GmbH, Tuebingen, Germany; 2 Rippin Consulting, Solingen, Germany; 3 Universitaetsklinik Tuebingen, Tuebingen, Germany

## 

Recently, the RNActive technology has been developed to generate highly active cancer vaccines. The antigen of choice is encoded by a sequence engineered mRNA that increases antigen expression up to 5 orders of magnitude. Immunogenicity of the RNActive vaccines is ensured by a built-in adjuvant function based on complexation of half of the mRNA with protamine that leads to activation of toll-like receptor 7 (TLR7). 5 intradermal administrations of a mRNA vaccine encoding PSA, PSCA, PSMA, and STEAP (CV9103) were tested in phase I/IIa studies in 44 patients with castrate-resistant prostate carcinoma (PCA). The vaccine was well tolerated and induced antigen-specific immunogenicity assessed by ex-vivo assays against all encoded antigens independent of the HLA-profile of patients. The overall immune response rate in evaluable patients was 80 %, close to 60% of these reacted against multiple antigens. In the subgroup of 36 patients with metastatic CRPC a median overall survival of over 30 months was estimated whereas the predicted survival according to the Halabi nomogram was 16.5 months. Patients responding to at least one tumor antigen had a reduced risk to die during the observation period compared to immunological non-responders, although the Halabi-predicted survival of both groups was similar. Responses against multiple antigens seemed to correlate even more strongly with improved survival, with patients reacting against 3 antigens s showing the longest survival. The results of this clinical study demonstrated high immunogenicity paired with a good safety profile for this new prostate cancer vaccine. The apparent correlation of immunogenicity with prolonged survival despite unfavorable patient characteristics according to the Halabi-score suggests a therapeutic effect. CV9104, an advancement of CV9103 encoding two more antigens is currently tested in a randomized, placebo-controlled phase IIb trial with overall survival as primary endpoint. It appears that mRNA based vaccines offer an ideal basis to exploit the potential of nucleotide based cancer vaccines for the benefit of patients. Precise data will be presented at the meeting.

